# Design of Integrated Micro-Fluxgate Magnetic Sensors: Advantages and Challenges of Numerical Analyses

**DOI:** 10.3390/s22030961

**Published:** 2022-01-26

**Authors:** Nicolò Marconato

**Affiliations:** Department of Industrial Engineering, University of Padova, 35131 Padua, Italy; nicolo.marconato@unipd.it

**Keywords:** fluxgate, magnetometers, micro-sensor, numerical analyses, finite element model, optimization

## Abstract

Miniaturization and on-chip integration are major lines of research in many branches of science and technology developments, undoubtedly in sensor technology. Fluxgate magnetometers are very sensitive, and accurate magnetic sensors able to detect weak fields both AC and DC, which in recent years saw a great effort in minimizing their dimensions, weight, and power consumption. The physics behind the fluxgate principle is rather complex and makes simulations difficult and only partially used in the literature. The limited physical access to micro sensors for measurements and the need to optimize the entire integrated system, including the sensor geometry and the excitation and readout circuits, make numerical analyses particularly useful in the design of miniaturized sensors. After a thorough review of the miniaturized solutions proposed so far, the present paper examines in detail the possibility of adopting a model based approach for designing miniaturized fluxgate sensors. The model of the fluxgate effect of two different technologies proposed in the literature has been implemented to benchmark simulation results with real data. In addition to the advantages for an optimized design, the implementation and computational challenges of the numerical analyses are precisely outlined.

## 1. Introduction

Magnetometers are sensors that allow for measuring external magnetic field and electric currents flowing into a conductor. The ability to measure these two basic quantities makes them essential instruments in a wide range of applications. A broad selection of magnetic sensors exploiting different physical principles have been developed, which differ in sensitivity, measurement range, cost, etc. Magnetic sensors based on the Hall and magnetoresistance effect are the most widespread, together with the so-called Fluxgate Sensors (FS), which are exceeded only by SQUIDs in terms of resolution. Their high sensitivity, high accuracy, temperature stability, and robustness [1,2,3,4,5] make them one of the best candidate to be applied in many fields such as astrophysics and satellite attitude control [6,7,8,9], geophysics [10], electronic compasses [11,12,13], current sensors [14,15], non-destructive testing [16,17], bio-medical diagnostics [18,19], etc. In recent years, particular efforts have been spent in miniaturizing and integrating FSs in digital chip-sets, paying particular attention at minimizing the power consumption, with the aim of developing light, portable, and flexible devices. Due to the rather complex working principle behind FSs, modeling and simulations of the operation of a fluxgate sensor are quite difficult and for this reason and are only partially adopted in the literature; generally, the design of FSs has been carried out mainly by prototyping and testing activities [20,21]. The design of miniaturized FSs would, instead, greatly benefit of numerical analyses: on the one hand, because of the limited accessibility for performing measurements on small prototypes and, on the other hand, the possibility to carry out precise optimization by conducting a thorough evaluation of the performances as function of the various parameters in order to maximize sensitivity and linear range, while minimizing power consumption and noise without the need to produce expensive and time consuming prototypes prior to design finalization. The present paper aims to analyze in detail the possibility of using numerical simulation as a design tool for miniaturized FS, evaluating advantages and limits of their usage. In Section 2, the operation principle of fluxgate sensors and the different developed configurations are summarized. The miniaturized solutions proposed in literature are reviewed in Section 3, highlighting advantages and drawbacks of the different solutions and the technologies (Section 3.1) and materials (Section 3.2) adopted. The typical issues affect the performance parameters of FSs and the complexities in correctly modeling the fluxgate effect are outlined in Section 4, thus consistently drawing out the essential features influencing the performance. A detailed review of available information on Co-based amorphous magnetic alloys is provided in Section 4.2. Section 4.3 and Section 4.4 describe in detail the models implemented in COMSOL Multiphysics in order to assess the fluxgate effect of two example of miniaturized FSs published in the literature, a planar and a 3D miniaturized sensor, respectively, allowing the comparison of the results with real data. Finally, in Section 5 and Section 6, discussion and conclusion are drawn.

## 2. Working Principle and Configurations of Fluxgate Sensors

The working principle of FSs is based on the magnetic induction, as in the case of pickup coils, but FS allows measuring also low frequency AC and DC magnetic field exploiting the non-linear dependence of the magnetic permeability to the magnetic field intensity of ferromagnetic materials. In general, the voltage induced in a sensing coil is given by Faraday’s law:

(1) $$V_{in}=-\frac{\partial \Phi }{\partial t}=-\frac{\partial (NA\mu_{0}\mu_{r}H)}{\partial t}=-\mu_{0}N\left(\mu_{r}A\frac{\partial H}{\partial t}+\mu_{r}H\frac{\partial A}{\partial t}+AH\frac{\partial \mu_{r}}{\partial t}\right)$$

where $\mu_{0}$ is the vacuum magnetic permeability, and Φ is the magnetic flux linked to the coil of area *A* and number of turns *N* supposed to be immersed in a uniform media with relative magnetic permeability $\mu_{r}$ and uniform magnetic field intensity *H*. This formula clearly neglect the demagnetization effect. In Equation (1), the three possible contributions to the time variation of the linked flux are made explicit: The first term corresponds to the emf induced in a pickup coil by a time-varying field, the second term corresponds to the emf induced in sensors where the cross-section is time dependent, such as in rotating coil sensors, while the third term is responsible for the fluxgate effect exploited in FSs, where emf is produced by the variation in time of the magnetic permeability experienced by the detection coil. Clearly, while in the first case only time-varying fields can be detected, DC fields can be measured as well in the second and third case.

**Figure 1:**
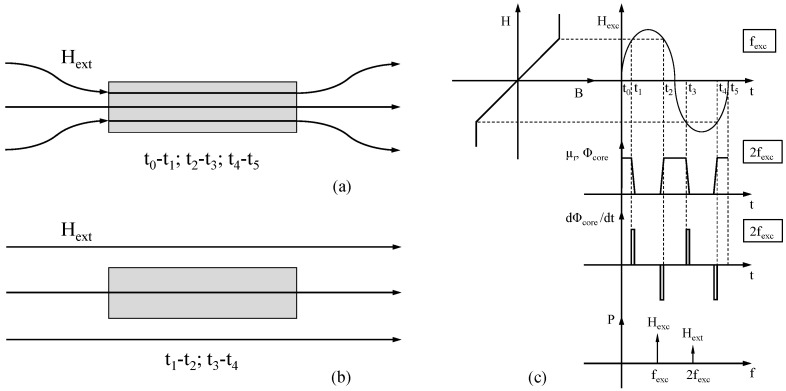
Scheme of the basic fluxgate working principle. (**a**) The core is not saturated, and the magnetic flux is concentrated inside. (**b**) The flux is “gated” out when the permeability drops to $\mu_{0}$. (**c**) Time evolution of the main quantities in a period of the excitation field $H_{exc}$.

As anticipated, FSs make use of a ferromagnetic core that is periodically saturated by the so-called excitation coil wound around the core, thus producing the required variation of the magnetic permeability experienced by the detection coil. This so-called magnetic chopping modulates magnetic permeability according to the core B-H loop, between $\mu_{0}$ and the maximum $\mu_{0}\mu_{r}$ value, with a periodic excitation field at a certain frequency. The magnetic flux is concentrated within the core when the material is not saturated and operates in the linear high permeability region of its B-H curve (Figure 1a), whereas it is chopped or “gated” out when it saturates and the permeability drops to $\mu_{0}$ (Figure 1b). When magnetic permeability abruptly changes, a pulse in the time derivative of the core magnetic flux $\Phi_{core}$ occurs, with amplitude proportional to the external field. This cycle occurs twice in each excitation period; thus, the information on the external magnetic field is included in the second (and higher even) harmonic component of the power spectrum of the flux change with time (Figure 1c).

As already mentioned, this principle allows detecting DC and low frequency magnetic fields. According to the Nyquist theorem, the excitation frequency must be at least two-times higher than the frequency of the external magnetic field to be detected. However, many times (10 to 100) larger excitation frequency are commonly adopted in practice. More specifically, the gating mechanism of a FS subject to a magnetic field H(t) with a detection coil of *N* turns having a cross-sectional area $A_{core}$, assumed equal to that of the ferromagnetic core, produces a voltage $V_{in}$ across the sensing coil provided by the change in time of the magnetic flux passing through the core cross section according to the following.

(2) $$V_{in}=-N\frac{\partial \Phi_{core}}{\partial t}=-\mu_{0}\mu_{r}\left(H\left(t\right)\right)NA_{core}\frac{\partial H(t)}{\partial t}-A_{core}H\left(t\right)\frac{\partial \mu_{0}\mu_{r}\left(H\left(t\right)\right)}{\partial H(t)}\frac{\partial H(t)}{\partial t}$$

The magnetostriction of magnetic cores adopted for FSs is generally negligible and the second contribution to the last term of Equation (1) can, thus, be neglected. The magnetic field H(t) consists of two components $H_{exc}\left(t\right)$, the excitation field produced by the periodic current flowing in the excitation coil(s), and $H_{ext}\left(t\right)$, the external magnetic field to be measured.

### Fluxgate Sensor Classification

**Figure 2:**
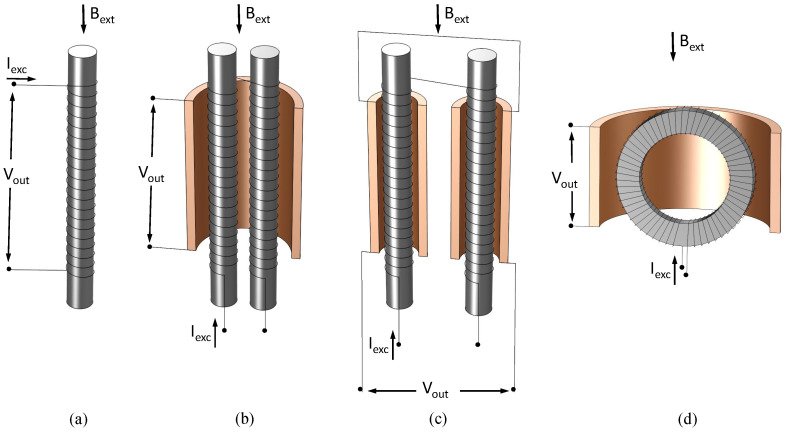
Parallel fluxgate configurations. (**a**) Single-core, (**b**) Vacquier, (**c**) Förster, (**d**) Aschenbrenner, and Goubau (ring-core); in gray is the ferromagnetic core and the detection winding is in orange when separate from the excitation.

**Figure 3:**
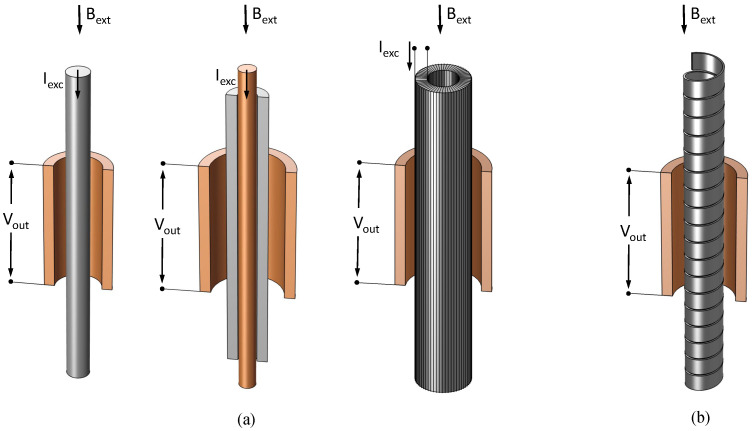
Orthogonal ((**a**) Aldredge) and mixed-mode ((**b**) Schonstedt) fluxgate configurations; in gray is the ferromagnetic core, and detection winding and solid excitation conductor are in orange (if present).

A first classification of FSs is according to the relative direction of the excitation and external magnetic field: the so-called parallel (Figure 2) and orthogonal (Figure 3) configurations. In the former, external and excitation field are in the same direction. The simplest configuration makes use of a single ferromagnetic core and two coils wound around of it. The so-called excitation coil is required to periodically saturate the core by an AC current, and the other is used as a sensing coil to detect flux changes throughout the core. The signal detected by the sense coil in this configuration has a large component on the first harmonic of the excitation frequency (first term of Equation (2)). This is eliminated (or at least highly reduced) in the so-called Vacquier or Förster configurations, where excitation fields in opposite directions are produced in two separate cores by properly wound excitation coils. A single sensing coil is wound by embracing both cores in the Vacquier type, and two separate sensing coils properly wound around each core and connected in series are instead adopted in the Förster one. In both cases, the flux generated by the excitation coils is subtracted and, thus, not detected by the sensing coils, except for a little amount due to manufacturing imperfections. The cores are also more easily saturated than in the single core version thanks to the high permeability return path for the excitation magnetic field provided by the second core. The saturation is clearly further eased in a completely closed core configuration, known as the ring-core sensor. In this configuration, the excitation coil is wound around a toroidal core and the sensing coil, wound so as to embrace the entire ring, and detects the magnetic field parallel to the core plane. Alternatively, a racetrack-shaped core with excitation coils separately wound around the long legs and the sense one embracing both is adopted [1].

Orthogonal FSs, where the excitation and external fields are perpendicular to each other, have two main configurations, as shown in Figure 3, known as Aldredge types. In the first configuration, the excitation is obtained by passing a current through the ferromagnetic core itself, producing a circular magnetic field in the cross-sectional plane of the core. The second configuration is similar to the ring core parallel type, with a circular core having the main dimension along the thickness of the ring so as to form a tubular shape and the excitation coil wound toroidally around it. Both configurations are sensitive to magnetic fields along the length of the ferromagnetic core, and the sensing coils are wound around the core length to detect the signal. Schonstedt proposed a mixed mode sensor [1] with a helical ferromagnetic core wound with a certain angle around the axis of the magnetic field to be measured. The excitation is again provided by the current passing through the helical core and sensing coil is wound around the core (Figure 3b).

In the following, for simplicity, only the gating mechanism for parallel configurations will be taken into account. In this case, the magnetic field across the core can be written as follows.

(3) $$H\left(t\right)=H_{exc}\left(t\right)+H_{ext}\left(t\right)$$

As already mentioned, the external field may also be a function of time, but it is possible to assume $H_{ext}$ as DC since excitation is selected at a much higher frequency than that of the external field.

## 3. Miniaturized Fluxgate Sensors

The early FSs, developed as high sensitivity, high resolution, and high accuracy magnetic sensors mainly for astrophysics and geophysics applications [6,7,8,9,10], had large and heavy sensor heads with solid ferromagnetic core and high turn number copper wire-wound coils, focusing on precision at the expense of weight and power consumption. Since more than two decades ago [22], several miniaturized and integrated solutions have been proposed, aiming at minimizing dimensions, weight, and power consumption without overlooking the objectives of high sensitivity, wide linear range, and low noise. In accordance with the geometry of the coils, they can be divided into two main families: 3D type and planar type. The former, called microsolenoid fluxgate sensors (MSFS) hereinafter, make use of a thin ring [23] or rectangular/racetrack shaped magnetic core (ring-type) [20,24,25,26,27,28] or two (Vacquier-Förster-type) [14,22,29] cores with micro-machined or PCB 3D coils wound around the thin cores. In [30], a double-axis microsolenoid FS is described, which implements a Förster-type sensor on the two leg pairs of a closed square shaped core. Some applications use single-core type, as [31], overlooking performances in place of other characteristics. The plane type family of micro FSs make use of planar coils instead (called planar coil FS (PCFS) hereinafter), with single [32,33] or double layer [34,35] straight core stripes for single-axis sensors, one cross-shaped core [36,37,38] for double-axis sensors, or a particular evolution of this last configuration for a three-axis sensor [39]. Hybrid solution have also been proposed, as in [40], where excitation coils are wound while detection coils are planar and use two cores in Förster-type configuration. All of them adopt the ‘second harmonic’ operation principle by the use of a lock-in amplifier to obtain second harmonic signal outputs by detection coils. A third miniaturized solution can be classified as orthogonal FSs and make use of a 3D solenoid pick-up coil and a meander-shaped thin core that carries the excitation current [41], operated in ’fundamental’ mode.

### 3.1. Technologies of Miniaturized Fluxgate

The coils and the core of miniaturized FSs are manufactured with PCB technology [23,25,27,33,35,38,39,40,41], allowing the construction sensors of several tens of millimeters or with different CMOS-compatible MEMS techniques [20,22,24,26,28,29,30,32,36,37], such as sputtering, electroplating, lithography, electron-beam evaporation, etc., which allow for achieving a further reduction in dimensions up to few millimeters. In [31], a conventional micro-printing technique is, instead, adopted to produce a mechanically flexible sensor.

Closed [20,23,24,25,26,27,28] or almost closed [34,35,38] magnetic path for the excitation field allows much deeper saturation of the sensor core, thus reducing the perming effect (the appearance of an offset shift when a FS is exposed to a magnetic shock), hysteresis, noise, and power consumption. Moreover, better performances in terms of sensitivity are obtained with closed-core configuration [20,23,24]. The double layer configurations proposed by [35,38] allows fair sensitivity in a quite limited range and at the expense of power consumption and planar dimensions. Moreover, the configurations described in [20,23] assure low noise, and the latter also has very limited power consumption (no data in this respect is given in [23]). The case of [14] deserves a separate discussion. Their FS exhibits fair sensitivity and an extended linear range despite the Vacquier-type configuration, thanks to the compensation operation in closed-loop. Very small dimensions and power consumption are achieved, with good noise immunity and thermal stability. The elongated shapes of the two cores implies a small demagnetization factor and a high number of coil turns, which permits a small excitation current peak required to fully saturate the core. This, together with the pulsed excitation, allows an efficient operation at high frequency, despite the high number of coil turns. In addition, the particular form factor is well suited for the presence of a compensation coil, which, together with the high operation frequency, allows a wide linear range and a good sensitivity, respectively. It can be said that a simple geometry was chosen at the expense of rather complex electronics. However, the very small size of the sensor requires an advanced manufacturing process. It is worth noting that this is the only sensor among those here cited that appears to be commercially available, and it is considered to be the 1D sensor with overall best performances. The sensor described in [22], which is one of the first miniaturized solution and first CMOS integrated device, adopted a similar design with lower performance in terms of linear range and noise.

Miniaturized FSs generally have only a limited number of turns of the pickup coil; thus. the sensitivity is lower than that of wire-wound fluxgate. For this reason, higher excitation frequencies are usually adopted, typically in the range of 100–500 kHz compared to few tens of kilohertz (kHz). On the other hand, excitation coils have a low number of turns; thus, large excitation current peaks are required to reduce the perming effect and improve the range of linear response. The quality factor of the excitation coil is generally low with respect to that of large-size FSs because of the small cross-sectional area of the magnetic core. For this reason, tuning the excitation circuit is, therefore, limited, complex, and not always possible. A possible alternative to reduce power consumption is the use of short excitation current pulses instead of the sine or square wave [14,27].

PCFS produced by CMOS-compatible technology can easily be integrated in an integrated interface circuit digital device [32,36,37]; however, present technology allows the integration of MSFS, and several examples already exist [14,22,29,30].

### 3.2. Ferromagnetic Materials Adopted in Miniaturized Fluxgate

It is well known that very high permeability and low saturation field ferromagnetic material are the best choice for FSs (together with low coercivity, low magnetostriction, and high electrical resistivity). Indeed, the higher the permeability and the lower the saturation field, the lower the excitation current required to fully saturate the core. Co-based amorphous alloys, also known as metallic glasses, and 80%Ni-20%Fe permalloy, are the best options for core material of micro FS thanks to their high permeability, low saturation field, small hysteresis, fast frequency response, and almost zero magnetostriction. There are two amorphous alloy commercially available known with the trademark of VITROVAC 6025 [25,27,31,33,35,36,38,42], produced by Vacuumschmelze, and Metglas 2714A [20,35,37,38,39,40,41,43] produced by Metglas Inc., Conway, United States. They are produced and supplied in ribbons with a thickness of 25 μm and of 15–20 μm, respectively, and have similar characteristics. Permalloy is generally electroplated [22,23,24,26,28,30,34] or sputtered [29] on a substrate during the MEMS process. The ferromagnetic cores in [32] consist of a NiFeMo alloy deposited on top of the finished industrial CMOS process with a simple postprocessing sequence at room temperature. Co-based amorphous alloys are generally adopted in PCB technology solutions and applied as cast, except for [44] where a 1 µm layer of VITROVAC 6025X was sputtered. In [37], the authors declare that they previously thinned down the ribbons to 10 μm by using a mechanical–chemical process to improve the sensitivity of the sensor and the saturation of the ferromagnetic core. However, the first statement has no theoretical basis, and it is not further analyzed there. The core thickness is indeed crucial in defining the characteristics of FSs. As already mentioned, since the induced output voltage is proportional to the area of the pickup winding, increasing the magnetic core’s thickness will proportionally improve the sensitivity of the sensor, but the limiting factor is demagnetization. Indeed, the thinner the core, the lower the demagnetization [45] and, consequently, the easier the saturation, with clear advantages in terms of power consumption, linear range and perming. On the other hand, the authors in [46,47] proved that larger core cross-sectional areas have beneficial effects in reducing the noise. Therefore, there is importance in defining the core dimensions and shape for achieving the required performances, maximizing some of them rather than others. In this respect, the advantage of electroplating over sputtering is that it allows an easier control of the thickness and pattern to desired dimensions and shape; thus, the sensitivity and the operational range can precisely be tailored.

It is worth noting that the requirements of FS cores, and consequently the adopted materials, induce power losses in the core in the frequency range of interest, generally negligible with respect to power losses in the winding [48].

## 4. Modeling in Design of Miniaturized FS

Numerical models are essential tools in designing an FS, which allows the process to speed up and reduce the costs related to the prototyping activity. It is possible to build electromagnetic models of a device providing precise correlation between particular geometries, with a given number of coil turns and core characteristics, and sensor performances. Finite element model (FEM) magnetostatic analyses allow optimizing the geometry in order to minimize the required excitation current able to saturate the core and, thus, to produce the fluxgate effect. Examples are reported in [26,33,35,38,39,40]. Time-domain transient FEM analyses are required instead in order to evaluate the fluxgate effect, as performed in [26,33,38]. Few information on the adopted electromagnetic models and, in particular, on the modeling of the core material is given in the cited works, which is instead considered essential to correctly estimate real sensor performances. In [29], a Simulink^®^ model of the response of an ideal FS is mentioned and not further described in the text, which presumably takes only the actual geometry of the object partially into account. Clearly, circuit-type models for the design of the excitation and read-out circuit can be implemented, as for example reported in [32].

The possibility to implement a behavioural circuit model taking into account the actual nonlinear inductance of the excitation coil allows for a model-based tuning of the excitation circuit in order to generate a peaked excitation current and, thus, improving the efficiency of the sensor.

In the next sections, the complexities in the development of a numerical model for the design of a miniaturized fluxgate are pointed out. For the purposes of this research, the described models were implemented in COMSOL Multiphysics^®^. Adopted formulations, constitutive relations, and constraints are natively supported features of COMSOL’s Magnetic Fields interface of the AC/DC Module [49], which includes also the Jiles–Atherton vector hysteresis model. Two cases of miniaturized FSs published in the literature, a PCFS [33] and an MSFS-type magnetometer [25], have been taken as reference to reproduce their experimental results with the implemented models. Before providing a detailed description of the models implemented for simulating the fluxgate effect of the mentioned real FSs, the main challenges that must be faced, both in terms of computational complexity and of required information on the core magnetic properties in order to obtain realistic results, are drawn in Section 4.1 and Section 4.2, respectively.

### 4.1. Challenges and Assumptions in Modelling Miniaturized Fluxgate Sensors

As already mentioned, neglecting noise considerations at this stage, general specifications in designing a miniaturized FS include the minimization of the sensor size and power consumption, while maximizing sensitivity and linear range. FEM codes are presently the best option for the numerical electromagnetic model simulating the actual response of a fluxgate sensor and, thus, carrying out a parametric optimization.

The first complexity lies in the inherent need of a fully 3D geometrical model for reproducing the fluxgate effect, for both PCFS and MSFS type sensors. As a matter of fact, the periodic summation and subtraction of the external excitation field alternatively in the two legs of the ferromagnetic core makes this an inherently three dimensional problem. Moreover, for modeling PCFSs, there are no symmetries that can be exploited to reduce the dimension of the problem, whereas, in case of MSFSs with particularly regular geometry, one forth of the entire object might be modeled while introducing a fairly minor error. Linked to this is the fact that an extremely fine mesh with a very high number of elements is required because of the extremely small thickness of the thin core and normally due to the conducting paths as well, compared to the overall dimensions of the object.

As already mentioned, preliminary magneto-static analyses can be useful to identify the minimum excitation current able to completely saturate the core. In any case, we have to deal with analyses in which the contribution of an external magnetic field and the one due to an excitation current distribution are superimposed, and the entire object is in presence of non-linear ferromagnetic materials.

In order to reproduce the fluxgate effect, in principle a time dependent analyses able to take into account the hysteresis behavior of the core has to be run. As already mentioned, it will be described in the next sections. Comsol multiphysics^®^ allows taking into account the actual hysteresis loop of ferromagnetic material by a five-parameters implementation of the Jiles–Atherton model [49,50]. However, in the case of material with an extremely narrow hysteresis loop, such as those under consideration, a small error is made by using the simpler model that considers the sole B-H curve, neglecting the magnetic hysteresis. A further simplification is the possibility of neglecting the effects of magnetic induction, given the extremely limited dimension orthogonal to the magnetic flux of the objects involved, the frequency range usually adopted and the relatively high electric resistivity of the core (about 10^−6^ Ωm).

A proprietary code implementing a filamentary version of a Partial Element Equivalent Circuit (PEEC) model [51] was used to verify that neglecting capacitive effects is also allowed, as described in Section 4.5.

The minimum time frame to be simulated corresponds to an entire period of the excitation current in the case of non-hysteretic analyses and 5/4 of the period in case the Jiles–Atherton model is adopted, in order to catch the effects of possible geometry and a lack of symmetry that is generally present.

The above makes the estimation of the fluxgate effect, for a single value of the external field, a very demanding analysis, both in terms of required memory and computation time. Evidently, in order to create the typical curve in the plane with the sensor output voltage ($V_{out}$) in the ordinate and the external magnetic field ($B_{ext}$) in the abscissa, a sequence of such analyses has to be run for different values of $B_{ext}$. The analyses described in the following, where a direct solver was chosen for the solution of the system of equations in order to speed up as much as possible the calculation, required about 60 GB of RAM and a day for a single analysis on a 16-core computer and, therefore, several days to reconstruct the entire $V_{out}-B_{ext}$ characteristic. All this makes proceeding straight to correct models capable of correctly reproducing the essential phenomena essential by adopting the correct formulations and solver settings and, thus, avoiding possible errors and minimizing the required efforts, which is one of the objectives of this work.

In addition to the problems described above related to the physical and numerical model to be adopted, the correct representation of the ferromagnetic core characteristic is an essential issue. Information on the magnetic characteristic of the materials of interest, 80% Ni-20% Fe permalloy and Co-based amorphous alloy, is scarce in the literature, particularly on their actual hysteresis cycles.

The choice of the two selected cases of PCFS and MSFS sensors to be modeled, in addition to the specific peculiarities that will be discussed in detail in Section 4.3 and Section 4.4, respectively, is justified by the fact that they both use VITROVAC 6025 as the core material, which is also the one that is relatively better documented. In Section 4.2, the available information and relative sources found on Co-based amorphous alloys most commonly employed in miniaturized FSs, with particular enphasis on the VITROVAC 6025, are reported.

### 4.2. Documented Information on the Hysteresis Loop of Co-Based Amorphous Alloy

As already mentioned, two “families” of Co-based amorphous alloys are suitable for the design of FSs are VITROVAC 6025, produced by Vacuumschmelze, and Metglas 2714A, produced by Metglas Inc. The peculiarity of amorphous metals is that they are soft magnetic material while being mechanically hard. In addition, these particular alloys are characterized by very high permeability and low saturation fields.

VITROVAC 6025 is provided with round (R), flat (F) or squared (Z) shaped hysteresis loop. In [42], it is also stated that saturation can vary between 0.53 and 0.59 T. Typical value of 0.55 T is generally adopted in literature. The variant VITROVAC 6025 X can be used without heat treatment. Finally, VITROVAC 6080/6070 are modified versions of the 6025 with higher saturation fields of 0.6 or 0.62 T, high permeabilities, and excellent linearity behavior.

Analogously, Metglas 2714A is provided with different hysteresis loop shapes depending on heat treatment [43]: Round loops can be achieved when annealing is carried out in the absence or with a transverse magnetic field, whereas square loops results from annealing in the presence of a longitudinal field. In this case, a saturation field of about 0.57 T and very small coercivity well below 0.01 A/m is obtained. Unannealed Metglas 2714A results in broader loops with lower saturation fields and higher coercivity.

**Figure 4:**
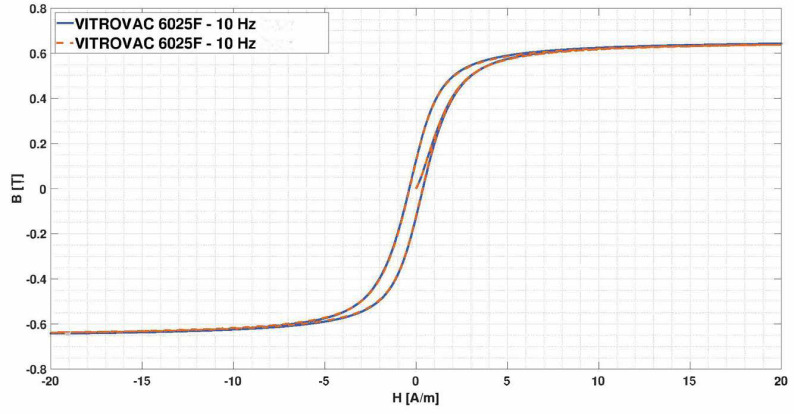
Hysteresis loops reconstructed from the two sets of Jiles–Atherton parameters reported in Table 1: continuous blue line from [52] and dashed orange line from [53], respectively.

For the purpose of this research, we searched for any studies published in the literature about identification of Jiles–Atherton parameters of VITROVAC 6025, and only the study of [52,53] was found. In [52], an identification technique, based on a genetic algorithm, has been applied to identify the hysteresis loop of a ring core of VITROVAC 6025F at 10 Hz. In [53], a different identification technique has been adopted on the same measured data provided in [52]. The Jiles–Atherton parameters obtained in the two studies are summarized in Table 1, and the reconstructed loops are reported in Figure 4, which shows that the two curves are almost overlapping.

**Figure 5:**
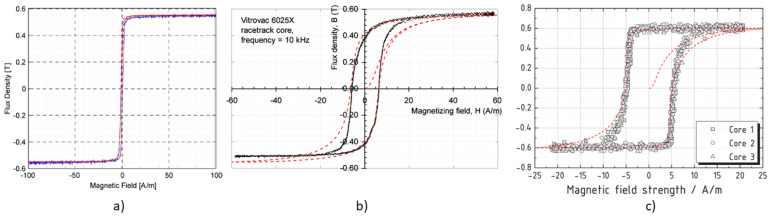
Fitted hysteresis loops (dashed red lines) overlapped to the original images for the cases named JA1, JA2, and JA3, respectively, in (**a**–**c**).

**Figure 6:**
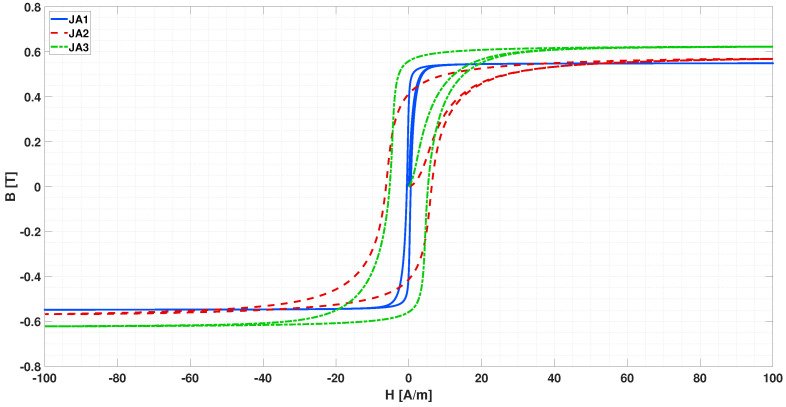
Comparison of the hysteresis loops reconstructed from the Jiles–Atherton parameters of fitted curves JA1 (continuous blue), JA2 (dashed red), and JA3 (dash-dotted green) reported in Table 2.

Given the rather high saturation field above 0.6 T, these Jiles–Atherton parameters have not been adopted, except for some tests, as described later. The magnetic characteristics adopted in this research were instead extracted by a manual fitting of the hysteresis curve reported in [44], in [27,54], starting from the parameters of Table 1. In particular, the curve reported in Figure 4 of [44], the curve in Figure 1 of [27] and the curve in Figure 6 of [54] have been fitted. In the following, these will be referred to with the codes JA1, JA2, and JA3, respectively, for convenience. The fitted curves JA1, JA2, and JA3 overlapped in the original images are shown in Figure 5a–c, respectively. The relevant Jiles–Atherton parameters for the different cases are shown in Table 2, and the comparison of the three curves can be seen in Figure 6.

In [27], it is stated that the JA2 curve was taken at 10 kHz, in the other cases instead the measurement frequency of the hysteresis loop is not specified. Presumably JA1 corresponds to the DC curve, since it constitutes data provided by the manufacturer VAC and because of the very narrow cycle, and JA3 corresponds to 5 kHz, since this is the driving frequency adopted in [54]. It is worth noting that these are only conjectures; however, they are reasonable as hysteresis loops generally become wider as frequency increases.

### 4.3. Modeling of a Planar Type Fluxgate Sensor

As already mentioned, the models developed for this research were implemented in the AC/DC Module [49] of Comsol multiphysics^®^, taking two real cases from the literature as references. First, the PCFS type sensor described in [33] was considered.

It is worth mentioning that in [33] a detailed description of the planar excitation and sensing coils geometries, as well as of the core and mutual distances, is given, which allowed an accurate reproduction of the geometrical model. As for the magnetic material characteristic, VITROVAC 6025X, $\mu_{r}\approx$ 100,000 and saturation induction $B_{s}=0.55$ T were adopted. As described in Section 4.2, in [44], the same authors reported the measurement of a VITROVAC 6025X hysteresis loop supplied from Vacuumschmelze, which was used as reference for the core characteristic in the magnetic model here described.

**Figure 7:**
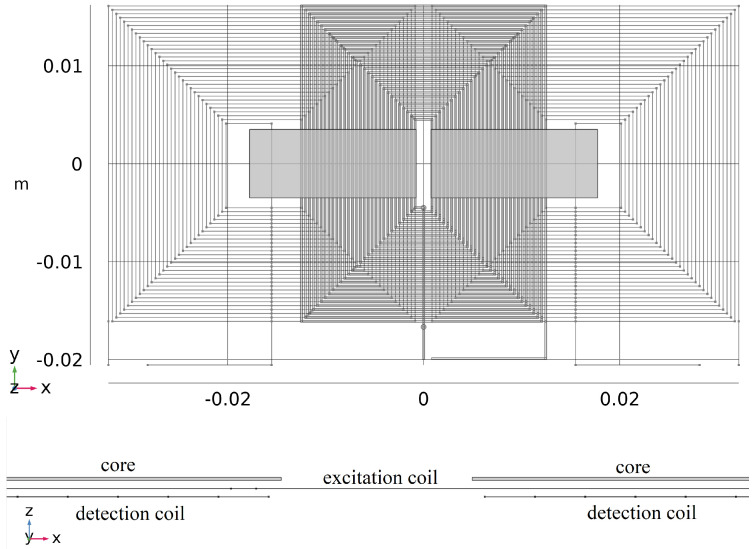
Implemented geometrical model reproducing the PCFS type sensor described in [33] Copyright 2022 IEEE Transactions on Instrumentation and Measurement; top view on the top and zoomed side view at the bottom of the figure.

In the model, the excitation coil is a surface body reproducing the real rectangular shaped 30 turns spiral coil with 400 μm pitch and zero thickness; the two sensing coils are implemented as filamentary rectangular-shaped spiral coil corresponding to the axis of the real coils (each one again with 30 turns and 400 μm pitch); and the two cores are solid bodies of rectangular shape with sizes of 17 × 7 mm^2^ and 25 μm thickness. Overall dimensions are 64 × 31 mm^2^. An image of the implemented geometrical model is shown in Figure 7.

The modeled excitation and sensing coils are placed in two planes along the z direction at a distance of 73.5 μm in order to take into account the mutual distance and thicknesses indicated in [33]. The distance between the cores and the excitation coil was chosen to be 65 μm assuming again that an insulating layer of 50 μm is interposed between them. Total thickness of the active elements in the model, from the top core surface to the sensing coil, is 155 μm

**Figure 8:**
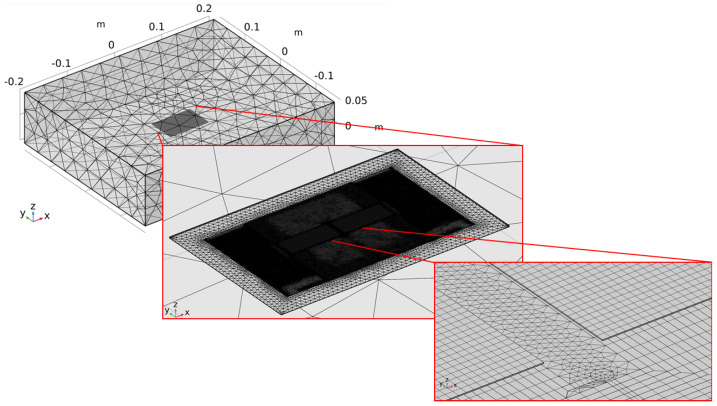
Mesh implemented for the PCFS model: the overall dimension of the air volume and different levels of detail (in red boxes) are shown.

Some box domains were also introduced for helping the creation of a good mesh. The critical points in the mesh creation are the core thickness of only 25 μm and the vertical distances between the excitation and sensing coils and between excitation coil and cores. A mapped mesh was created were possible, which is in the core volumes and on the excitation coil surface, in order to reduce the number of elements while maintaining a good solution there. The transition to free-tetrahedral mesh in the narrow space between the different layers was another critical point, solved by a proper positioning and dimension of the additional boxes. A mesh of 1,617,587 volume elements on the whole was obtained. In Figure 8, different levels of detail of the implemented mesh are shown.

**Figure 9:**
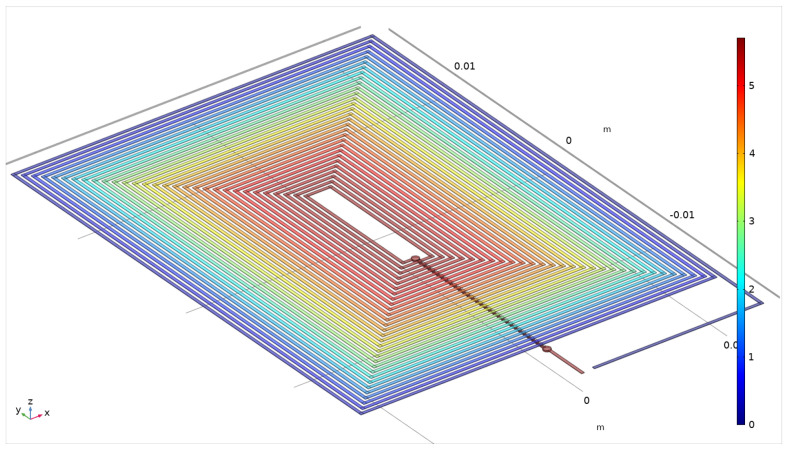
Calculated voltage distribution for the surface current model, expressed in V.

First step of the analysis is the determination of the static distribution of the current density in the excitation coil. Given the planar geometry, the “Electric Currents in Shells” interface of the AC/DC Module is adopted. The current density distribution due to a 1 A current injected on one terminal, with the other one grounded, is calculated. In Figure 9, the obtained voltage distribution is shown.

The Magnetic Fields interface of the AC/DC Module is used for the magnetic model, where the “Reduced Field” option is chosen in the Background Field setting in order to apply an external field. The “External Magnetic Vector Potential” node is then added to actually apply this boundary condition on the external boundaries of the air domain. The “Gauge Fixing for A-field” node is also added in order to correctly solve the problem with direct solvers. Indeed, the node imposes the divergence of the magnetic vector potential (**A**) to be equal to zero everywhere, gauge condition required for magneto-static analysis implementing the **A** formulation in order to have a uniquely determined solution and, therefore, a non-singular numerical problem. A “Surface Current Density” node is added in order do apply the excitation current as the forcer of the magnetic model. Here, the previously calculated current density distribution is used as input by multiplying each vector component for a properly defined analytic function reproducing the sinusoidal waveform. Analyses were carried out with a sinusoidal driving current with an amplitude of 700 mA and frequency of 10 kHz, and external magnetic field coplanar to the FS and parallel to the ferromagnetic cores. Linear type element as FEM discretization, and constant type Newton methods for the nonlinear solution were chosen in order to obtain convergence. Pseudo time-stepping and Anderson acceleration were added for stabilization and acceleration of the solution. In addition, a preliminary magneto-static solution with the only forcing external field, that is, with zero excitation current, is useful for the convergence of the following transient analysis by using the previous solution as the initial condition.

**Figure 10:**
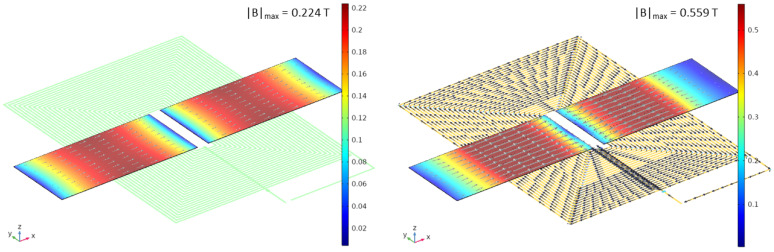
Contour-plot of the magnetic field norm on the cores with gray arrows indicating the field direction, together with contour-plot of the current density norm on the excitation coil with black arrows indicating the current direction, at t=0 s (**left**) and t=25 μs (**right**) corresponding to zero and the peak current, respectively, for a 150 μT external field. Color bars in T.

The contour-plot of the magnetic field norm on the cores, together with its direction (gray arrows), is reported in Figure 10 for two time instants, one corresponding to zero current (t=0 s) and the other to the current peak (*t* = 25 μs), on the left and right side of the figure, respectively, when a 150 μT external field is applied. Black arrows show the excitation current direction.

The output voltage is obtained by the time derivative of the difference of the flux linked to the two pick-up coils. The fluxes are calculated as the line integral of the tangential component of the vector magnetic potential along the coils. Given the squared geometry of the coil, line integration is easily obtained when considering *x* and *y* components of the vector potential along the segments in the same direction. The operation is more complex in case of generic 3D coils, as it will be described in the next paragraph.

**Figure 11:**
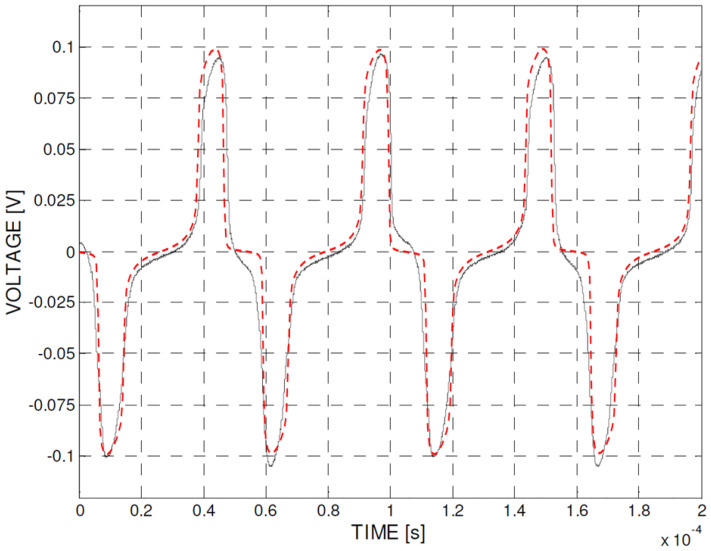
Calculated differential output voltage with 150 μT external field (dashed-dotted red line) overlapped with the experimental results from Figure 13 in [33] (Copyright 2022 IEEE Transactions on Instrumentation and Measurement) obtained in the same conditions.

A first, and analysis was carried out with 150 μT external field and a non-hysteretic B-H characteristic of the core extracted from the JA1 loop. The result of this transient magnetic simulation in terms of differential output voltage is shown in Figure 11 (dashed-dotted red line), overlapped to the experimental voltage from Figure 13 in [33] obtained in the same conditions. Simulation shows very good accordance with the experimental results.

**Figure 12:**
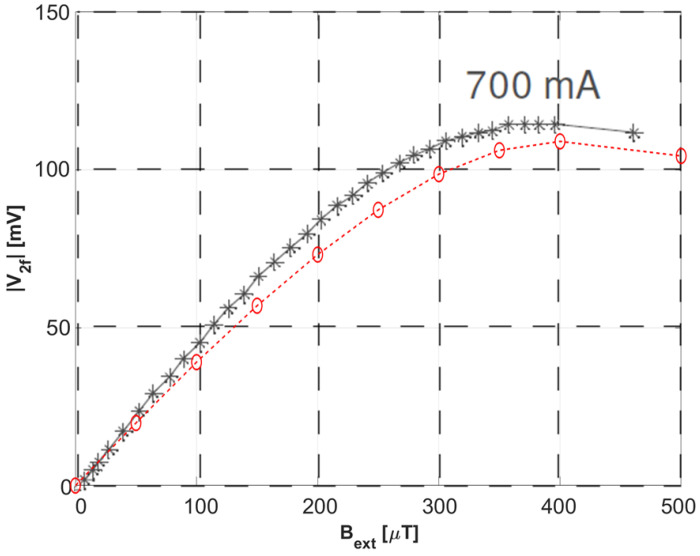
Second harmonic of the calculated differential output voltage plotted against the related external magnetic field (dashed red line and circles), overlapped to the experimental results from Figure 14 in [33] Copyright 2022 IEEE Transactions on Instrumentation and Measurement.

The same analysis was then repeated for different values of the external magnetic field in a range from 50 to 500 μT. The second harmonic of the several differential voltages obtained is then calculated in MATLAB^®^ by numerical fast Fourier transform (FFT). The values of the second harmonic content of the output voltage are plotted against the related applied external field in Figure 12, again overlapped with the experimental results shown in Figure 14 from [33].

**Figure 13:**
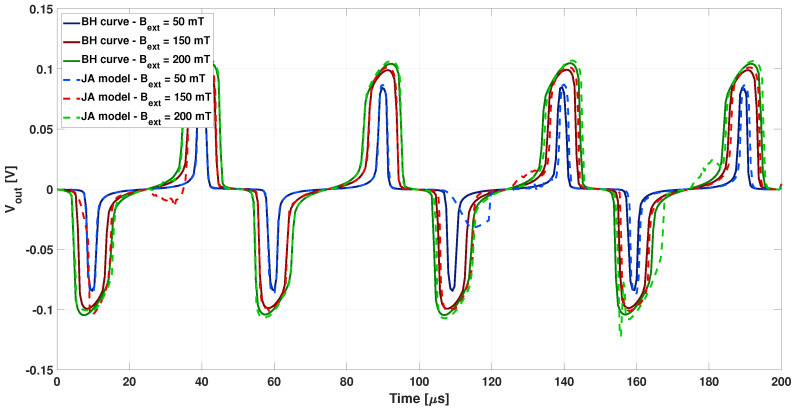
Differential output voltage calculated by adopting the parameters related to the JA1 loop (dotted lighter lines), for three values of the external magnetic field (50, 150, and 200 μT), compared to the related ones calculated with the corresponding BH characteristic (continuous darker lines).

Simulations are in good agreement with experimental data and the model developed is considered able to estimate the behaviour of the sensor with a good level of detail even by neglecting the hysteretic feature of the core. How the second harmonic is obtained in the two cases might justify the discrepancy between simulation and experimental data of Figure 12. However, the influence of considering realistic hysteretic loops has been tested. The analysis was repeated by adopting the Jiles–Atherton model of core magnetization. The differential output voltage waveform obtained by adopting the parameters related to the JA1 loop (dotted lines) for three values of the external magnetic field (50, 150 and 200 μT) are shown in Figure 13, compared to the related results obtained without hysteresis (continuous lines).

On one hand, a slight higher amplitude of the output voltage is observed in Figure 13 when the hysteresis is considered. On the other hand, the picture shows also that numerical instabilities occur when the Jiles–Atherton model is adopted.

**Figure 14:**
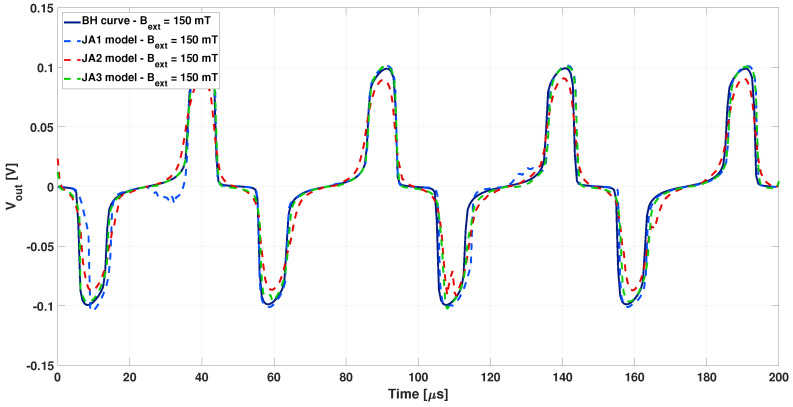
Differential output voltage calculated adopting the Jiles–Atherton model for the JA1, JA2, and JA3 loops (dotted lighter lines) with external magnetic field of 150 μT), compared to the one calculated without hysteresis (continuous darker lines).

Finally, the same analysis was repeated adopting the JA2 and JA3 parameters of the Jiles–Atherton model, applying an external magnetic field of 150 μT. The comparison with the previous cases at the same external field is reported in Figure 14.

A smoother variation from the positive to the negative peaks of the output voltage is observed in Figure 14 with larger and smoother hysteresis loop (JA2 and JA3 loops). Moreover, the numerical issues encountered when the Jiles–Atherton model is applied seemed to be significant the narrower and the steeper the hysteresis loop was.

### 4.4. Modeling of a 3D Type Fluxgate Sensor

The miniaturized fluxgate magnetometer described in [25] was considered in order to test the capability of modeling MSFS sensors. This particular design was chosen for three main reasons:

- Due to the geometry with markedly 3D features;
- For the use of VITROVAC 6025 as core material;
- To numerically investigate the influence of the air gap.

It must be said, however, that unlike the previous case where the data provided on the geometry and the material used were quite detailed, in this case, information was rather limited.

**Figure 15:**
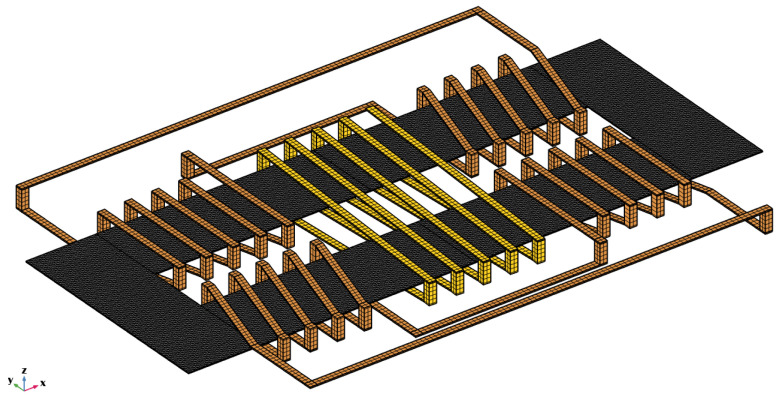
Meshed model implemented in Comsol^®^ reproducing the MSFS sensor described in [25] Copyright 2022 Elsevier; in gray is the ferromagnetic core, the excitation coils are in orange, and the detection coil is in yellow.

The sensor consists of a thin racetrack core made of not well specified VITROVAC 6025: two excitation coils and one detection coil. In this case, all the components were modelled as volume bodies. A picture of the implemented model is shown in Figure 15, where the mesh of the active components is reported.

The core is 15.9 mm in length, 6 mm wide, 25 μm thick, and the legs are 2.5 mm wide. The coil trace are 200 μm wide and 35 μm thick, and there is a distance of 200 μm separating the upper traces from the core and of 300 μm from the bottom one for a total thickness of 595 μm.

A similar approach as the one previously described is adopted for building the mesh, creating a mapped mesh where possible (core and coils), and a box body was added to divide the air volume into a smaller one with refined mesh and the outer with a coarser mesh.

As for the previous case, a preliminary electric conduction analysis is performed for a 1 A input current. In this case, the Electric Current module is used to calculate the volume current density distribution inside the excitation coils. Again, the resulting distribution is used to drive the magnetic analysis, by multiplying it for a sine wave analytical function at 10 kHz and 150 mA amplitude, corresponding to the driving current adopted in [25].

The flux linked to the detection coil is again obtained by the line integral of the magnetic vector potential tangential to the coil. However, because of the 3D coil geometry, which is not along Cartesian coordinates, this operation is not as straightforward as for the planar case. A third analysis is introduced, carried out with the “Curvilinear Coordinate” module, in order to find a vector field of unitary norm flowing inside the detection coil domain and, thus, provides a vector base to define point-by-point the component of the magnetic vector potential tangential to the coil by the scalar product of the two vector fields.

**Figure 16:**
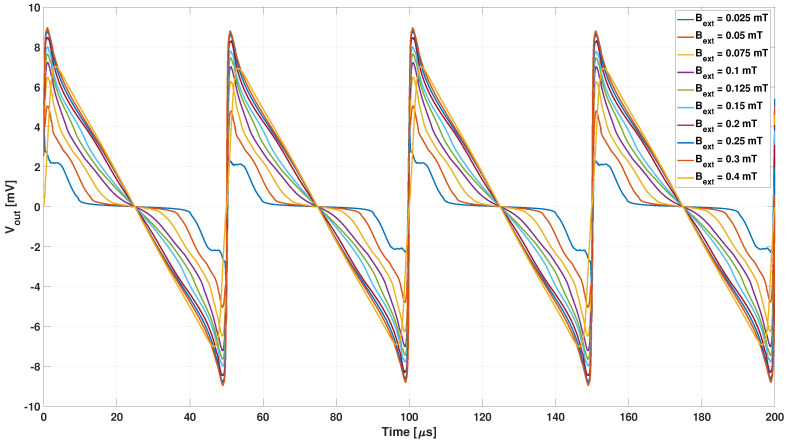
Output voltage obtained with the non-hysteretic B-H curve from JA1 material model for different values of the external magnetic field.

The analyses carried out aimed at reproducing the relation between the second harmonic of the output voltage as a function of the external field shown in Figure 3 of [25]. A first analysis was carried out with the non-hysteretic B-H curve obtained by the JA1 core material model. The time trend of the output voltage for different values of the external magnetic field is shown in Figure 16.

**Figure 17:**
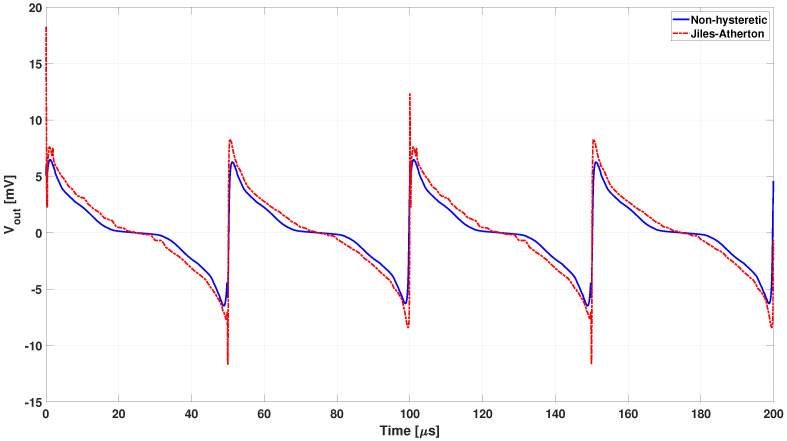
Output voltage obtained with the hysteretic Jiles–Atherton model with the JA1 parameters for an external magnetic field of 100 μT (red dashed-dotted line), compared to the one calculated in the same condition with non-hysteretic B-H curve (solid blue line).

The same analysis was then carried out by adopting the Jiles–Atherton model with the parameters of the JA1 hysteresys loop. Again, in this case, the numerical solution is affected by a certain degree of inaccuracy. The output voltages related to an external magnetic field of 100 μT calculated with the hysteretic and non-hysteretic magnetic characteristics are compared in Figure 17.

**Figure 18:**
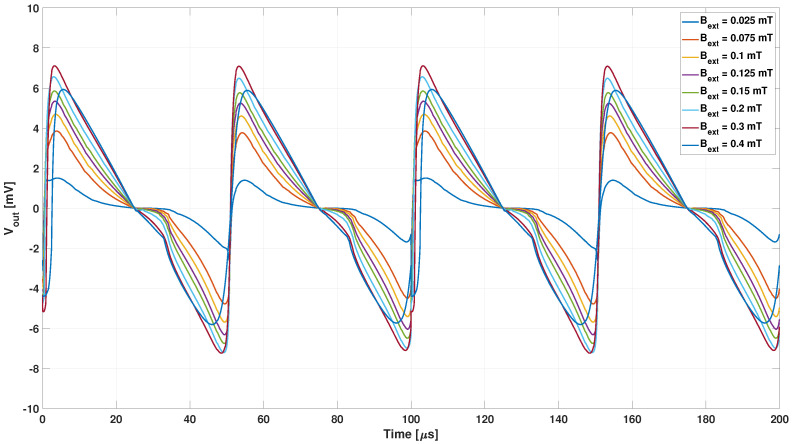
Output voltage obtained with the hysteretic Jiles–Atherton model with the JA2 parameters for different values of the external magnetic field.

The analysis taking into account the JA2 hysteresis loop was also carried out for the entire range of external magnetic field values from 25 to 400 μT, as reported in Figure 18, where the lower harmonic content is clear.

Different magnetic field values were tested with the JA3 hysteresis loop, obtaining convergence problems for most of them.

**Figure 19:**
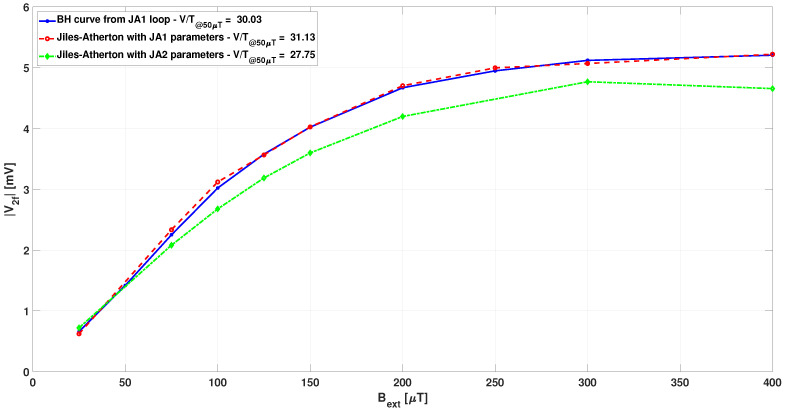
Second harmonic of the output voltage against the related external magnetic field for the different analyzed cases: non-hysteretic JA1 model (solid blue), hysteretic JA1 (dashed red), and JA2 (dahed-dotted green) model.

The second harmonic of the output voltage for the different analyzed cases has been obtained by the FFT of the calculated signals, and they are plotted in Figure 19 against the related external magnetic field.

From Figure 19, it is clear that the effect of hysteresis is negligible when very low coercivity materials are considered, as expected. A non negligible, although limited, difference is observed when considering material with coercivity of about 5 A/m, as it is more reasonable at frequencies higher than some kilohertz. The obtained curves of the calculated second harmonic of the output voltage do not precisely match the results from [25]: The sensitivity results are about 30% higher, and the linear range is slightly lower. Nevertheless, the use of numerical analyses here presented is considered useful in carrying out design optimization, for example, maximizing the linear range rather than sensor sensitivity. In addition, it is worth noting that FS operated in closed loop generally have better performances. In this case, a numerical approach is useful to start a sensor design oriented from the very beginning to this operation mode, allowing design optimization both in terms of sensitivity and compensation field uniformity.

**Figure 20:**
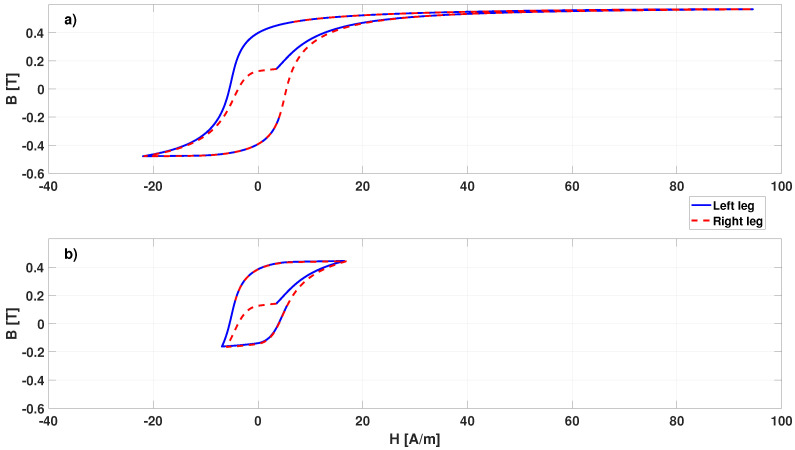
Hysteresis loop covered in one excitation period by the left (solid blue lines) and right (dashed red lines) core legs with the closed core configuration (**a**) and with a 0.5 mm air gap (**b**), when an external field of 100 μT is applied, and the JA2 hysteresis model parameters are used.

The analysis was then repeated by introducing a 0.5 mm air gap on both the short legs of the core, as described in [25]. In this case, no fluxgate effect is obtained with the model for external field up to more than 100 μT. All magnetic characteristics were tested, both considering and neglecting the hysteresis, and similar results were obtained in all cases. Indeed, a very small air gap increases very much the reluctance of the magnetic circuit. The calculations show that for an external magnetic field up to about 100 μT, the core does not reach saturation. The hysteresis loop covered in one excitation period by the central cross section in the two long legs of the core (in average) with the closed core configuration, when an external field of 100 μT is applied, is shown Figure 20a). This was obtained by integrating the magnetic field intensity and the magnetic field density on the cross section of the core in the middle of the two long legs, divided by the area of the involved surface. Due to the external magnetic field, the loop is asymmetric, and the starting point does not correspond to the point at H=0 and B=0. In addition, because of the opposite excitation field in the two legs, with respect to the external field, the loop is covered starting from the same point (when $I_{exc}=0$), but in opposite direction in the two legs. The same was calculated in the case of open core with a 0.5 mm gap and shown in Figure 20b), where it is clear that saturation is not reached. This picture refers to data obtained with JA2 hysteresis parameters, but similar results are obtained in the other cases.

In order to saturate the core at low external field value with air gap, a higher current would be required or a magnetic material with similar permeability and saturation field of about 0.4 T, which does not appear to be possible.

### 4.5. Excitation Frequency Response

A further investigation was carried out in order to estimate the presence of capacitive contribution on the excitation coil impedance in the frequency range of interest. This has been conducted with a proprietary code implementing the partial element equivalent circuit (PEEC) method described in [51], which allows the integral solution of electromagnetic fields, considering at the same time conductive and capacitive effects at low and high frequency. This is particularly suited in our case for estimating the equivalent impedance of winding at different frequencies. We adopted the criterion that, up to the frequency where the imaginary part of the impedance divided by the angular frequency is equal to the low frequency value, the capacitive term is negligible and the purely magnetic solution calculated in Comsol is valid.

**Figure 21:**
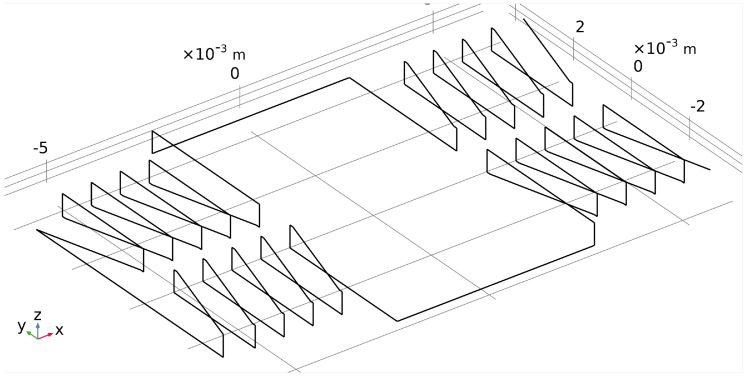
Filamentary model of the excitation oil used to calculate its frequency response.

**Figure 22:**
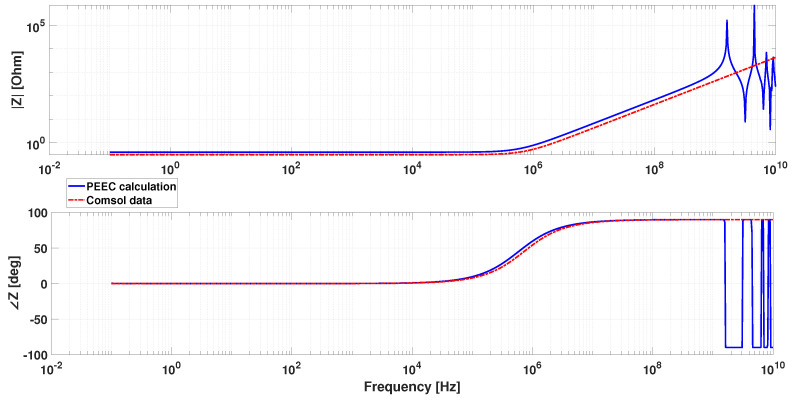
Frequency response of the excitation coil impedance calculated with the PEEC filamentary model (solid blue line) and with resistance and inductance parameters estimated in Comsol (dashed-dotted red line).

The frequency response of the winding has been calculated on the basis of the filamentary model shown in Figure 21, which corresponds to the central axis of the solid path of the model shown in Figure 15. A volume formulation of the method is also possible [55] but less suitable for the particular application. In addition, it is worth noting that in this case the calculation was carried out in air, i.e., without the ferromagnetic core. Indeed, the aim of this simulation is only to assess the capacitive contribution on the equivalent winding impedance in the frequency range of interest, which is considered independent of the presence of the core. The method might take into account magnetic media [56], but in this particular case it is considered more useful to verify the frequency behaviour of the air winding impedance alone. Therefore, the result of this analysis has been compared to the inductance estimated with the Comsol model described above in absence of the ferromagnetic core. The inductance calculated in Comsol is equal to 0.067 µH, whereas the one calculated as an imaginary part of the impedance from the PEEC model divided by the angular frequency, in the frequency range where it is constant, is equal to 0.106 µH. The main point is that this value remains constant up to about 100 MHz. The amplitude and phase of the equivalent impedance as a function of the frequency calculated with the PEEC code are shown in Figure 22, together with the one calculated from the resistance and inductance values estimated in Comsol. This assessment has, thus, proven the validity of estimations carried out in Comsol in the frequency range of interest.

It is worth noting that this particular FS configuration has a flat impedance up to very high frequency, but other geometries, with more compact winding, can have a much narrow response that can limit the use at high excitation frequency. This method can, thus, be useful to fix limits in the operational range in relation to the particular geometry implemented.

## 5. Discussion

The first part of the present work reports a review of the state of the art on miniaturized FSs design, where a comparison of the performance of the different technologies documented in the literature is reported, which results in affirming the superiority of MSFSs over PCFSs in terms of both higher performances (sensitivity, linear range, noise, etc.) and power consumption.

For this reason, the main focus of this research was the assessment of the predicting capability of FEM magnetic analyses for the estimation of the fluxgate effect of MSFS-type miniaturized fluxgate sensors.

A review on the use of numerical modeling applied so far in this field has also been carried out, as well as a review of published work with real data on the magnetic characteristics of Co based amorphous alloy VITROVAC 6025.

The analyses carried out for this research adopted realistic hysteretic loops. The results obtained have been also compared with the case where the simpler non-hysteretic B-H relation between the magnetic induction and magnetic field intensity is used. The comparison results in the conclusion that the use of the non-hysteretic B-H characteristic is certainly sufficient for an initial optimization of the design, and results are very close when using ferromagnetic material with coercivity lower than some A/m.

The proposed model was first successfully validated with numerical and experimental data reported in [33], reproducing the PCFS-type sensors here described, by using the non-hysteretic material model. Then, modeling of an MSFS-type magnetometer was tested, taking as reference the design reported in [25]. In this case, the results are not perfectly matching, but the overall behavior is correctly reproduced. The reason of discrepancy can be several factors:

- The geometry implemented possibly does not accurately reproduce the actual design, which is worth noting that it is not described in detail in [25];
- The real paths of the conductive traces connecting the excitation coil to the power supply probably are quite different from what simulated;
- Possible differences in the magnetic characteristic.

However, in this regards no significant differences were expected compared to the magnetic properties adopted in this work. Indeed, in private communication with manufacturer Vacuumschmelze, it was confirmed, as reported in [42], that the minimum residual field that can be obtained is limited to 0.53 T for the particular alloy used in VITROVAC 6025.

Certainly it is not possible to contest the results in [25] related to the operation when an air gap is introduced with the proposed numerical analysis. Nevertheless, in our opinion, introducing air gaps in order to change sensitivity/linear range is not an appropriate option, and it is rather preferable to act on driving frequency and current amplitude (and/or turn number). In addition, in the case of a 1 mm gap, the authors’ experimental test also shows that, with an external magnetic field of up to 75–100 μT, no fluxgate effect is observed. Therefore, a sensor operating in this condition could not be properly used, at least in the low field range.

Numerical modeling allows optimizing sensor design, particularly when the following objectives are pursued:

- Find the best trade off among excitation current, frequency, and coil turns for the particular application, by considering the following in general:
  – The higher the current, the wider the linear range, but the higher the power consumption;
  – The higher the frequency, the higher the sensitivity, but the higher the supply voltage for given currents;
  – The higher the coil turns, the lower the required current and, thus, the power consumption, but inductance becomes higher, which limits the frequency and, thus, the sensitivity and bandwidth;
- Maximize the core saturation for minimizing perming and noise.

The models here presented proved to be effective in reproducing the fluxgate effect in different possible configurations, and it is believed that they are useful in pursuing all the above objectives, including assessing the degree of saturation in different configurations and operating conditions, as well as determining the demagnetisation factor of the core configuration considered. The influence of the lack of symmetry of the detection coil is also observed. However, numerical optimization is a time-consuming activity due to the long calculation time required for necessary analyses. In addition, minimum computational resources are required. At least 10 core CPU, 60 GB ram memory, and about 1 TB for storage are suggested.

As already mentioned, good results can be obtained by also neglecting magnetic hysteresis when a material with coercivity lower than a few ampere per meter is adopted. More precise results can be achieved by adopting the Jiles–Atherton hysteresis model, but it is worth noting that this is computationally more complex, and it suffers from numerical instability when very steep hysteresis cycles are implemented. Indeed, it was proven in the numerical experiment carried out that the particular shape of the hysteresis loop influences the computation of the Jiles–Atherton model: Wide and gradual hysteresis loops are easy to calculated, whereas narrow and steep loops suffer convergence (thus, requiring more time when convergence is achieved) and solution stability problems. It is worth noting that the particular configuration also affects the applicability of the Jiles–Atherton model: indeed, no considerable instability was experienced when solving the PCFS type model with the JA3 magnetic model, whereas almost no results could be achieved with the same material for the MSFS model.

A PEEC model was adopted to estimate the frequency range where the excitation coil can correctly work.

Before concluding, some considerations about FS noise can be useful. A major factor in determining the noise level of FSs certainly is the core material. It is mainly due to Barkhausen noise, but the lowest possible magnetostriction is certainly important in minimizing noise. Nevertheless, noise power seems to be affected by total core losses, as well as lower saturation field, in addition to receiving benefits in terms of linear range and power consumption, which implies lower fluxgate noise [57,58]. Connected to this is the fact that higher excitation peaks decrease the noise level by allowing the entire core volume to saturate. In addition, a higher excitation frequency is generally beneficial up to a certain limit corresponding to a minimum noise. However, the particular core geometric configuration is also important, since the demagnetization factor determines the contribution of material noise to the effective noise affecting the sensor output [2] and because the higher surface area of the magnetic material, the higher the fluxgate noise power [59]. While it is well known that fluxgate sensitivity depends on the curvature of the B-H curve, i.e., on its second derivative [59,60,61], although presumably sharper curvature implies higher noise, it is currently not possible to assert this with much certainty. All of that said, even though numerical analyses do not allow implicitly taking into account noise effect, it is possible to set an optimization oriented to achieve a low effective noise level by considering the previous remarks.

Future research will focus on a thorough characterization of Co-based magnetic alloys, taking into account the different grades available, with realistic geometry of the testing devices of interest for miniaturized sensor design.

## 6. Conclusions

An in-depth review of the state of the art of fluxgate microsensor technology is reported, showing the advantages and drawbacks of the different solutions implemente, and the superior performance of microsolenoid type sensors rather than the planar ones.

Different magnetic characteristics of realistic Co-based amorphous magnetic materials (VITROVAC 6025) have been numerically tested, both considering simple B-H non-hysteretic curve and by applying the Jiles–Atherton hysteretic model of the material, showing the limits of the two approaches.

An example of the two miniaturized fluxgate sensor topologies, a microsolenoid and a planar type sensor, was simulated in Comsol^®^ and successfully benchmarked with experimental data from the literature, with the aim of testing the possibility of using the numerical approach for a design optimization in terms of sensitivity, linear range, and power consumption.

Future work will aim at the characterization of Co-based amorphous alloy of different “classes” (annealing) from different producers in realistic geometries.

## Figures and Tables

**Table 1:** Jiles–Atherton parameters of a ring core of VITROVAC 6025F at 10 Hz identified by the methods described in [52] Copyright 2022 Elsevier and [53] Copyright 2022 AIP Publishing.

Parameter	Method [52]	Method [53]
α	$1.097\times 10^{-7}$	$0.51\times 10^{-7}$
*a*	0.594	0.580
*c*	0.664	0.626
*k*	0.416	0.401
Ms	$5.27\times 10^{5}$	$5.23\times 10^{5}$

**Table 2:** Jiles–Atherton parameters of the fitted curves JA1, JA2, and JA3.

Parameter	JA1	JA2	JA3
α	$8\times 10^{-7}$	$2.5\times 10^{-5}$	$1.12\times 10^{-5}$
*a*	0.16	4	1
*c*	0.664	0.1	0.1
*k*	0.716	7	6.9
Ms	$4.37\times 10^{5}$	$4.7\times 10^{5}$	$5\times 10^{5}$

## References

[1] Primdahl F. (1979). The fluxgate magnetometer. J. Phys. E Sci. Instrum..

[2] Ripka P. (1992). Review of fluxgate sensors. Sens. Actuators A Phys..

[3] Bol R., Overshott K.J. (2008). Sensors: A Comprehensive Survey. Volume 5, Magnetic Sensors.

[4] Tumanski S. (2013). Modern magnetic field sensors—A review. Prz. Elektrotechniczny.

[5] Ripka P. (2003). Advances in fluxgate sensors. Sens. Actuators A Phys..

[6] Miles D.M., Mann I.R., Ciurzynski M., Barona D., Narod B.B., Bennest J.R., Pakhotin I.P., Kale A., Bruner B., Nokes C.D. (2016). A miniature, low-power scientific fluxgate magnetometer: A stepping-stone to cube satellite constellation missions. J. Geophys. Res. Space Phys..

[7] Acuña M.H. (2002). Space-based magnetometers. Rev. Sci. Instrum..

[8] Nielsen O., Petersen J., Primdahl F., Brauer P., Hernando B., Fernandez A., Merayo J., Ripka P. (1995). Development, construction and analysis of the ‘OErsted’ fluxgate magnetometer. Meas. Sci. Technol..

[9] Musmann G., Afanassiev Y.V. (2010). Fluxgate Magnetometers for Space Research.

[10] Maire P.L., Bertrand L., Munschy M., Diraison M., Géraud Y. (2020). Aerial magnetic mapping with a UAV and a fluxgate magnetometer: A new method for rapid mapping and upscaling from the field to regional scale. Geophys. Prospect..

[11] Kaluza F., Grüger A., Grüger H. (2003). New and future applications of fluxgate sensors. Sens. Actuators A Phys..

[12] Park H., Hwang J., Choi W., Shim D., Na K., Choi S. (2004). Development of micro-fluxgate sensors with electroplated magnetic cores for electronic compass. Sens. Actuators A Phys..

[13] Baschirotto A., Dallago E., Malcovati P., Marchesi M., Venchi G. Precise vector-2D magnetic field sensor system for electronic compass. Proceedings of the SENSORS, 2004 IEEE.

[14] Snoeij M.F., Schaffer V., Udayashankar S., Ivanov M.V. An integrated fluxgate magnetometer for use in closed-loop/open-loop isolated current sensing. Proceedings of the ESSCIRC Conference 2015—41st European Solid-State Circuits Conference (ESSCIRC).

[15] Yang X., Li Y., Zheng W., Guo W., Wang Y., Yan R. (2015). Design and realization of a novel compact fluxgate current sensor. IEEE Trans. Magn..

[16] Wang H., Chen S., Zhang S., Yuan Z., Zhang H., Fang D., Zhu J. (2017). A high-performance portable transient electro-magnetic sensor for unexploded ordnance detection. Sensors.

[17] Grüger H. (2003). Array of miniaturized fluxgate sensors for non-destructive testing applications. Sens. Actuators A Phys..

[18] Tomek J., Platil A., Ripka P., Kašpar P. (2006). Application of fluxgate gradiometer in magnetopneumography. Sens. Actuators A Phys..

[19] Dolabdjian C., Saez S., Toledo A., Robbes D. (1998). Signal-to-noise improvement of bio-magnetic signals using a flux-gate probe and real time signal processing. Rev. Sci. Instrum..

[20] Lei C., Wang R., Zhou Y., Zhou Z.M. (2016). Reverse Optimization of an Integrated Solenoid Fluxgate Sensor Based on Co-based Amorphous Soft Magnetic Ribbon. J. Electron. Mater..

[21] Navaei N., Roshanghias A., Lenzhofer M., Ortner M. (2018). Analysis of Single- and Double Core Planar Fluxgate Structures. Proceedings.

[22] Gottfried-Gottfried R., Budde W., Jähne R., Kück H., Sauer B., Ulbricht S., Wende U. (1996). A Miniaturized Magnetic Field Sensor System Consisting Of A Planar Fluxgate Sensor and a CMOS Readout Circuitry. Sens. Actuators A Phys..

[23] Tipek A., O’Donnell T., Ripka P., Kubik J. (2005). Excitation and Temperature Stability of PCB Fluxgate Sensor. IEEE Sens. J..

[24] Liakopoulos T.M., Ahn C.H. (1999). A micro-fluxgate magnetic sensor using micromachined planar solenoid coils. Sens. Actuators A Phys..

[25] Dezuari O., Belloy E., Gilbert S., Gijs M. (2000). Printed circuit board integrated fluxgate sensor. Sens. Actuators A Phys..

[26] Wu P., Ahn C.H. (2007). A Fully Integrated Ring-Type Fluxgate Sensor Based on a Localized Core Saturation Method. IEEE Trans. Magn..

[27] Kubík J., Pavel L., Ripka P., Kašpar P. (2007). Low-Power Printed Circuit Board Fluxgate Sensor. IEEE Sens. J..

[28] Lei C., Sun X.C., Zhou Y. (2009). MEMS micro fluxgate sensors with mutual vertical excitation coils and detection coils. Microsyst. Technol..

[29] Delevoye E., Audoin M., Beranger M., Cuchet R., Hida R., Jager T. (2008). Microfluxgate sensors for high frequency and low power applications. Sens. Actuators A.

[30] Choi W., Kim J. (2006). Two-axis micro fluxgate sensor on single chip. Microsyst. Technol..

[31] Schoinas S., Guamra A.E., Moreillon F., Passeraub P. (2020). Fabrication and Characterization of a Flexible Fluxgate Sensor with Pad-Printed Solenoid Coils. Sensors.

[32] Kawahito S., Maier C., Schneiher M., Zimmermann M., Baltes H. (1999). A 2D CMOS microfluxgate sensor system for digital detection of weak magnetic fields. IEEE J. Solid-State Circuits.

[33] Baschirotto A., Dallago E., Malcovati P., Marchesi M., Venchi G. Fluxgate magnetic sensor in PCB technology. Proceedings of the 21st IEEE Instrumentation and Measurement Technology Conference (IEEE Cat. No. 04CH37510).

[34] Ripka P., Kawahito S., Choi S.O., Tipek A., Ishida M. (2001). Micro-fluxgate sensor with closed core. Sens. Actuators A Phys..

[35] Lenzhofer M., Ortner M., Schulz G., Stahr J. A New Approach For a Planar Miniaturized PCB Based High Sensitivity Fluxgate Sensor Design. Proceedings of the AMA Conferences 2017.

[36] Chiesi L., Kejik P., Janossy B., Popovic R. (2000). CMOS planar 2D micro-fluxgate sensor. Sens. Actuators A Phys..

[37] Drljača P.M., Kejik P., Vincent F., Piguet D., Gueissaz F., Popović R.S. (2004). Single core fully integrated CMOS micro-fluxgate magnetometer. Sens. Actuators A Phys..

[38] Baschirotto A., Dallago E., Malcovati P., Marchesi M., Venchi G. (2006). Development and comparative analysis of fluxgate magnetic sensor structures in PCB technology. IEEE Trans. Magn..

[39] Lu C., Huang J. (2015). A 3-Axis Miniature Magnetic Sensor Based on a Planar Fluxgate Magnetometer with an Orthogonal Fluxguide. Sensors.

[40] Lu C., Huang J., Chiu P., Chiu S., Jeng J. (2014). High-Sensitivity Low-Noise Miniature Fluxgate Magnetometers Using a Flip Chip Conceptual Design. Sensors.

[41] Zhi S., Feng Z., Lei C. (2019). Improved Performance of Fundamental Mode Orthogonal Fluxgate Using a Micro-Patterned Meander-Shaped Ribbon Core. Sensors.

[42] Vitrovac^®^ Product Literature, Vacuumschmelze, Hanau, Germany. www.vacuumschmelze.com.

[43] Metglas^®^ Product Literature, Conway, United States. http://www.metglas.com/tech/.

[44] Baschirotto A., Dallago E., Malcovati P., Marchesi M., Melissano E., Morelli M., Siciliano P., Venchi G. (2009). An integrated microfluxgate magnetic sensor with front-end circuitry. IEEE Trans. Instrum. Meas..

[45] Chen D., Pardo E., Sanchez A. (2005). Demagnetizing Factors for Rectangular Prisms. IEEE Trans. Magn..

[46] Jie F., Ning N., Ji W., Chiriac H., Xiaoping L. (2009). Study of the noise in multicore orthogonal fluxgate sensors based on Ni-Fe/Cu composite microwire arrays. IEEE Trans. Magn..

[47] Ripka P., Butta M., Jie F., Li X. (2010). Sensitivity and noise of wire-core transverse fluxgate. IEEE Trans. Magn..

[48] Korepanov V.E., Marusenkov A. (2012). Flux-Gate Magnetometers Design Peculiarities. Surv. Geophys..

[49] (2020). AC/DC Module User’s Guide. *COMSOL 5.5*. https://doc.comsol.com/5.5/doc/com.comsol.help.acdc/ACDCModuleUsersGuide.pdf.

[50] Jiles D.C., Atherton D. (1984). Theory of ferromagnetic hysteresis. J. Appl. Phys..

[51] Torchio R., Alotto P., Bettini P., Voltolina D., Moro F. (2018). A 3-D PEEC Formulation Based on the Cell Method for Full-Wave Analyses With Conductive, Dielectric, and Magnetic Media. IEEE Trans. Magn..

[52] Chwastek K., Szczyglowski J. (2006). Identification of a hysteresis model parameters with genetic algorithms. Math. Comput. Simul..

[53] Zaman M., Hansen P., Neustock L., Padhy P., Hesselink L. (2016). Adjoint method for estimating Jiles-Atherton hysteresis model parameters. J. Appl. Phys..

[54] Volkmar C., Geile C., Neumann A., Hannemann K. (2019). Direct-current current transformer for the measurement of an electric propulsion ion beam. Rev. Sci. Instrum..

[55] Torchio R. (2019). A Volume PEEC Formulation Based on the Cell Method for Electromagnetic Problems From Low to High Frequency. IEEE Trans. Antennas Propag..

[56] Torchio R., Moro F., Meunier G., Guichon J.M., Chadebec O. (2019). An Extension of Unstructured-PEEC Method to Magnetic Media. IEEE Trans. Magn..

[57] Shirae K. (1984). Noise in amorphous magnetic materials. IEEE Trans. Magn..

[58] Narod B.B., Bennest J.R., Strom-Olsen J.O., Nezil F., Dunlap R.A. (1985). An evaluation of the noise performance of Fe, Co, Si, and B amorphous alloys in ring-core fluxgate magnetometers. Can. J. Phys..

[59] Narod B.B. (2014). The origin of noise and magnetic hysteresis in crystalline permalloy ring-core fluxgate sensors. Geosci. Instrum. Methods Data Syst..

[60] Marshall S. (1966). Sensitivity of the ring-core magnetometer. IEEE Trans. Magn..

[61] Primdahl F. (1970). The fluxgate mechanism, part I: The gating curves of parallel and orthogonal fluxgates. IEEE Trans. Magn..

